# Debromination of Waste Circuit Boards by Reaction in Solid and Liquid Phases: Phenomenological Behavior and Kinetics

**DOI:** 10.3390/polym15061388

**Published:** 2023-03-10

**Authors:** Juan A. Conesa, Gerard Gandon-Ros, María F. Gómez-Rico, Ignacio Aracil

**Affiliations:** 1 Department of Chemical Engineering, University of Alicante, P.O. Box 99, E-03080 Alicante, Spain; 2 Institute of Chemical Process Engineering, University of Alicante, P.O. Box 99, E-03080 Alicante, Spain

**Keywords:** marble sludge, planetary ball mill, mechanochemistry, potassium carbonate, hydrothermal process, WEEE, electronic waste

## Abstract

The debromination of waste circuit boards (WCBs) used in computer motherboards and components has been studied with two different pieces of equipment. Firstly, the reaction of small particles (around one millimeter in diameter) and larger pieces obtained from WCBs was carried out with several solutions of K_2_CO_3_ in small non-stirred batch reactors at 200–225 °C. The kinetics of this heterogeneous reaction has been studied considering both the mass transfer and chemical reaction steps, concluding that the chemical step is much slower than diffusion. Additionally, similar WCBs were debrominated using a planetary ball mill and solid reactants, namely calcined CaO, marble sludge, and calcined marble sludge. A kinetic model has been applied to this reaction, finding that an exponential model is able to explain the results quite satisfactorily. The activity of the marble sludge is about 13% of that of pure CaO and is increased to 29% when slightly calcinating its calcite at only 800 °C for 2 h.

## 1. Introduction

It is well known that the development of electronic products has brought with it an increase in the use and production of brominated compounds, which act as flame retardants in these products [1,2,3] and can lead to the formation of toxic compounds during waste management treatments at the end of the electronic product’s life.

Various methods for the selective elimination of halogenated compounds from wastes, especially chlorinated and brominated ones, have been described in the literature [4,5,6,7]. The treatment methods that stand out are the use of not very high temperatures in the liquid phase, in the so-called hydrothermal carbonization (HTC) [8,9,10], and the solid phase reaction of the compounds containing halogens with specific reagents in highly energetic environments such as ball mills (i.e., horizontal mill, largely used for commercial purposes) and planetary ball mills (smaller but higher energy laboratory equipment) [11,12,13,14,15,16,17]. Other studies were focused on debromination processes based on the thermal decomposition of the materials [18,19]. Interesting studies on the debromination of different materials by mechanochemical degradation are also in the literature [20,21].

The interest of these dechlorination and debromination processes is centered on either having a material that can be used again [22] or preparing it for destruction in thermal treatment furnaces profiting from its energetic value [23], in which the formation of dioxin-like compounds can be more effectively controlled [24]. In both cases, the strategy would mean net energy savings and a very positive environmental balance.

In heterogeneous systems, the global kinetic expression must incorporate the different processes which involve both physical transport and reaction steps [25,26]. Frequently, one of the stages has the greatest contribution to the overall resistance: the slow or controlling stage, which can be considered as the only one that influences the rate (leading to simpler expressions [25]). The mass transfer must be considered so that in situations where this is the limiting factor, the driving forces are diffusion and phase equilibrium, while when the rate of reaction is the limiting factor, these forces are chemical equilibrium and its kinetics. In many real situations, however, both factors must be considered.

The kinetics of the transfer of halogenated compounds has been superficially treated in the literature, practically restricted to planetary ball mills. For this equipment, kinetic models have been described, with kinetic constants dependent on the energy applied to the reacting mix [27,28]. When studying the kinetics of heterogeneous reactions, what we try to find out is, first, what the rates of each of the processes involved are, both mass transfer and chemical reaction, and what the characteristic reaction constants of these are. With all this, the purpose of the kinetic study and modeling is to design a reactor that accomplishes the debromination in an optimal way and to obtain valuable products and/or energy. The present work aims to obtain a model that fits runs performed in different conditions and/or processes and with the correct treatment of the data.

## 2. Experimental Setup

### 2.1. Materials

WCBs used in the present work were metal-free FR-4 epoxy fiberglass substrates supplied by CISA (Circuitos Impresos S.A., Madrid, Spain). This WCB had a thickness of 1 mm and consisted of an overlapping of 5 laminates of cross-linked glass fiber immersed in epoxy resin with TBBPA as a flame retardant. A characterization of WCBs was performed by elemental analysis (27.5 wt.% C, 2.5 wt.% H, 1.1 wt.% N, and 24.6 wt.% O), ash content (44.3 wt.%), and bromine content determination (6.4 wt.%).

The marble sludge used in the planetary ball mill was provided by Mármoles Hermanos Jiménez (Alicante, Spain). The material was dried at 100 °C for 12 h. A calcined material was also prepared by heating at 800 °C for 2 h. Both materials were characterized. The elemental analyzer Thermo Finnigan Flash 1112 provided 15.46 and 12.33 wt% C content for sludge and calcined sludge, respectively, the rest corresponding to the ash content. XRF (Philips Magix Pro PW2400 equipped with a rhodium tube and beryllium window), and ICP-MS showed high Ca and Mg concentrations, which increased, as expected, after calcination. More details on the materials used can be found in previous studies [9,29].

### 2.2. Description of the Runs

**Figure 1:**
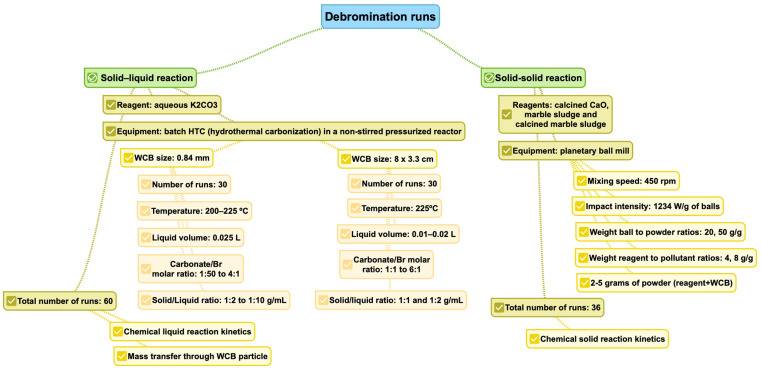
Scheme of the different runs performed for the kinetic analysis.

**Figure 2:**
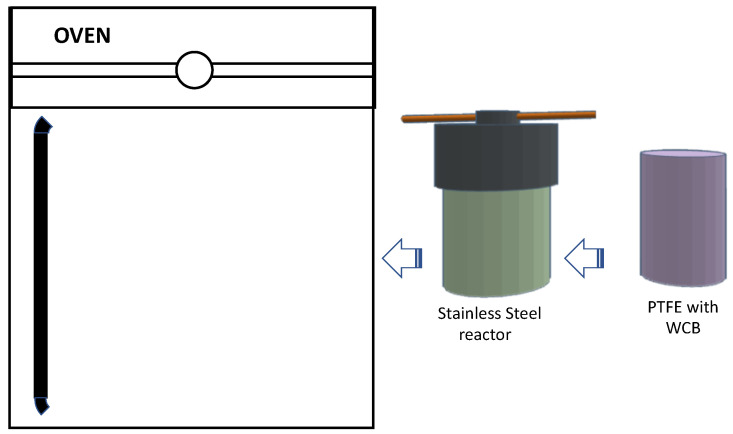
Scheme of the procedure for HTC batch runs.

For the debromination study, two different experimental equipment were used [9,29]. Figure 1 shows a scheme of the different runs performed. For the study of debromination in the liquid phase, 60 different runs were performed using solutions of potassium carbonate as active reagent and pressurized recipients reaching 200 or 225 °C. Half of these runs were performed using WCBs cut and shredded to small 0.84 mm particles, and the other half of runs were performed with pieces of 8 × 3.3 cm, approximately. This was performed to consider the role of diffusion of bromine through this material. In addition, two of the experiments had replicates. A scheme of this procedure is shown in Figure 2.

On the other hand, 36 solid-solid debromination runs were performed in a planetary ball mill, subjecting different reagent/WCBs weight ratios and ball-to-powder ratios to a 450-rpm rotation speed for different residence times. In these runs, a specific amount of WCB and the reacting media (CaO, marble sludge, or calcined marble sludge) were introduced in a mill where they were subjected to different milling programs. The planetary mill consists of one grinding jar, which is arranged eccentrically on a so-called sun wheel. The direction of movement of the sun wheel is opposite to that of the grinding jars. The grinding balls in the grinding jars are subjected to superimposed rotational movements. More details on the results of these runs can be found in previous studies [9,29].

## 3. Kinetics of Debromination

### 3.1. Debromination in Liquid Phase: K_2_CO_3_ as Active Agent for the Reaction

Specific conditions of the runs performed in the batch HTC reactor are shown in Table 1 (small particles) and Table 2 (larger pieces of WCBs), together with the result of the debromination efficiency (DE) achieved experimentally. DE was defined as the fraction of bromine content transferred from the solid to the liquid phase. The soluble bromine content was analyzed by ion chromatography (Metrohm, 850 ProfIC AnCat- MCS) after extracting a minimal amount of liquid in order to keep any alterations to the reaction medium in progress to a minimum. Actually, the debromination efficiencies (DE) were determined as if they were reached for each condition in an identical process with no extractions.

**Figure 3:**
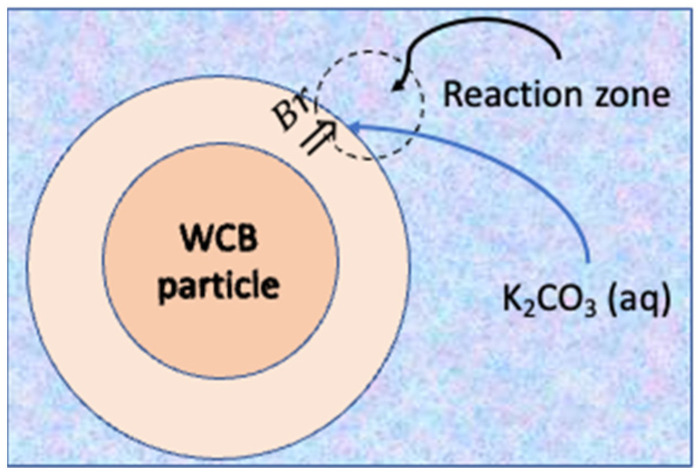
Reaction of bromine contained in WCB solid particles and aqueous reactants in the film surrounding surface of particles.

In the HTC process, during the debromination, a combined mechanism of mass transfer of bromine from the center of the particle to the surface and a chemical reaction occurs. For the bromine contained in the WCB to react with the potassium present in the liquid, two steps in a series must occur. Figure 3 shows a scheme of the process taking place.

The first step is the mass transfer of bromine from the center of the WCB particles to the reacting surface (under high-pressure conditions):

Br (in particle center) → Br (surface)

The rate of this process is given by the mass transfer coefficient in such a way that:

(1) $$\mathrm{BMTR}=k_{C}\left(C_{Bc}-C_{Bs}\right)\ldots g/\left(L\cdot s\right)$$

where BMTR is the bromine mass transfer rate from the center to the surface of the particle. $k_{C}$ is the (internal) mass transfer coefficient, and $\left(C_{Bc}-C_{Bs}\right)$ is the difference in the bromine concentration (in g/L) between the center of the particle (*C_Bc_*, approx. constant during the process) and the solid surface (*C_Bs_*, probably changing with time). Usually, the mass transfer coefficient varies approx. linearly with temperature, so:

(2) $$k_{C}=k_{C0}\cdot T$$

In this expression, *k_C_* is given in s^−1^ and *k_C_*_0_ in (s·K)^−1^.

The second step in the global process is the reaction between bromine species and potassium in the liquid phase, where the reaction rate can be expressed by:

(3) $$\mathrm{BRR}=kC_{K}C_{Bs}$$

BRR is the bromine reaction rate, that was hypothesized to be first-order with respect to the bromine and to the potassium concentration. In the prior expression, *C_K_* is the concentration of potassium (g/L) in the liquid (varying among the different experiments), and *k* is the kinetic constant (L/(g·s)) at the reaction temperature. The Arrhenius law can be applied so:

(4) $$k=k_{0}\cdot \exp\left(-\frac{E}{RT}\right)$$

Since mass transfer and the chemical reaction will debrominate the WCBs particles, the global rate for bromine elimination (*r_B_*) will be given by the contribution of both processes. In a process in which several consecutive steps are taken for the reaction to occur, as it is the one being analyzed, it is necessary to determine the rate at which the two processes occur separately. Generally, one of them is slower, and the final rate of the process will be equal to that of the stage whose rate is lower, bearing in mind that bromine cannot accumulate on the particle surface. In this sense, both mass transfer and reaction should be equal:

(5) $$r_{B}=\mathrm{BMTR}=\mathrm{BRR}=k_{C}\left(C_{Bc}-C_{Bs}\right)=kC_{K}C_{Bs}=\frac{\left(C_{Bc}-C_{Bs}\right)}{\frac{1}{k_{C}}}=\frac{C_{Bs}}{\frac{1}{kC_{K}}}=\frac{C_{Bc}}{\frac{1}{k_{C}}+\frac{1}{kC_{K}}}$$

This last equation will allow us to estimate which of the two stages is slower and, therefore, control the process by comparing the values of the two addends in the denominator of the expression [25]. If the mass transfer is much faster than reaction, *k_C_* >> *k*·*C_K_*, and Equation (5) will become $r_{B}=kC_{K}C_{Bc}$. On the contrary, if reaction is much faster than mass transfer, *k_C_* << *k*·*C_K_* and the rate velocity would be $r_{B}=k_{C}C_{Bc}$. In any case, expression shown in Equation (5) would be valid for calculating global rate.

In a batch reactor such as that used in the present study, the mass balance of the bromine contained in the liquid phase can be expressed by:

(6) $$\frac{dm_{B}}{dt}=V_{L}r_{B}\ldots \frac{g}{s}$$

where *V_L_* is the liquid volume and *m_B_* is the mass of bromine transferred to the liquid phase. From Equations (5) and (6), it can be written:

(7) $$\frac{dm_{B}}{dt}=V_{L}\left(\frac{C_{Bc}}{\frac{1}{k_{C}}+\frac{1}{kC_{K}}}\right)=\frac{m_{Bc}}{\frac{1}{k_{C}}+\frac{1}{kC_{K}}}$$

Subjected to the initial condition of *m_B_* = 0 for *t* = 0 and using the finite differences method (first order) [25], Equation (7) can be expressed as:

(8) $$\frac{m_{B}^{t+1}-m_{B}^{t}}{\Delta t}=\frac{m_{Bc}}{\frac{1}{k_{C}}+\frac{1}{kC_{K}}}$$

In the previous equation, *m_Bc_* refers to the amount (grams) of bromine in the center of the WCB particle being debrominated. For the calculation, a constant value of 4% of the mass of WCB introduced will be used, according to the results of the characterization of the material [9]. The superscripts ‘*t* + 1’ and ‘*t*’ refer to the values of the variables at different consecutive time values. Then:

(9) $$m_{B}^{t+1}=m_{B}^{t}+\left(\frac{m_{Bc}}{\frac{1}{k_{C}}+\frac{1}{kC_{K}}}\right)\cdot \Delta t$$

In this way, by choosing an adequately low value of time increment, the mass of bromine transferred to the liquid for given values of [*k_C_*_0_, *k*_0_, E/R] can be simulated, comparing the *DE* calculated for the final time in each run with the experimental one:

(10) $$DE_{calculated}\left(\%\right)=\frac{m_{B,final}}{m_{Bc}}\cdot 100$$

For the optimization of these three parameters, the following objective function is defined:

(11) $$OF=\sum_{all\;runs}^{}{\left(DE_{experimental}-DE_{calculated}\right)}^{2}$$

**Figure 4:**
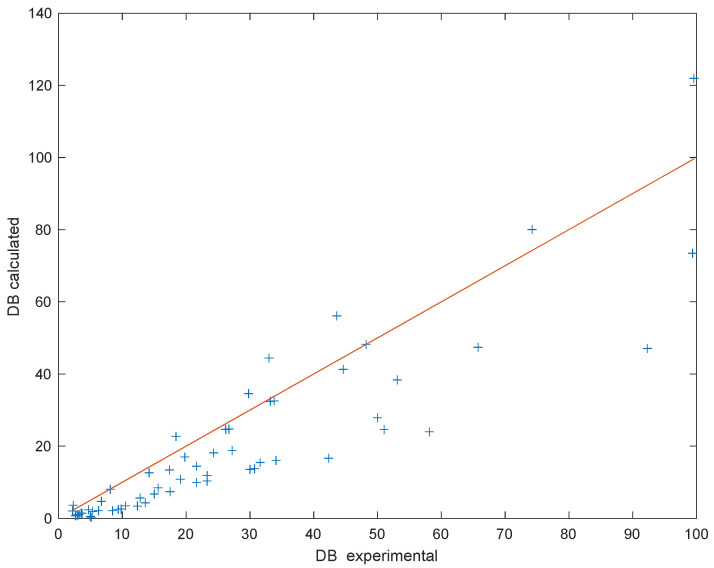
Results of the fitting of bromine transfer from solid WCB particles to reacting K_2_CO_3_ aqueous media.

The optimization of all 60 runs performed in this equipment, minimizing the *OF* defined in Equation (11), permitted the calculation of the kinetic parameters. For the optimization of the runs performed with different particle sizes, different values of *k_C_*_0_ and *k*_0_ were assumed, and a single value of E/R was used. Table 3 shows the results. The fitting obtained is shown in Figure 4 as *DE* calculated vs. experimental values. The coefficient of correlation calculated for this fit is R = 0.9444 (R^2^ = 0.8919).

From these values, we can compare the rates of both processes to elucidate the controlling mechanism. To compare reaction and mass transfer rates, it is necessary to evaluate the value of (*k·*C_Kmean_), where C_Kmean_ is the mean value of the concentration of potassium in the corresponding runs. Further, the value of *k_C_* should be calculated at the reaction temperature. Table 4 shows the corresponding values of the constants calculated at 225 °C.

As we can see, the mass transfer rate is much faster than the chemical reaction, with the controlling step being the last. If we compare the values of the constants optimized for the different particle sizes, we see that small particles promote both mass transfer and chemical reaction. Generally, it is expected that mass transfer from a large WCB particle would be more problematic than from previously ground particles. Nevertheless, if the mass transfer were very fast, these differences would not be observed. This is what happens in our system, where no great differences are observed in the rate of mass transfer from particles of very different sizes.

### 3.2. Debromination in Solid Phase: CaO and Marble Sludge Acting as Active Agent in a planetary Ball Mill

As commented above, the other equipment where the debromination has been carried out in a planetary ball mill [29]. In this equipment, debromination is produced during the grinding process of different quantities of mixtures of WCB and various reagents in the presence of a fixed quantity of steel balls. During the runs, performed at 450 rpm, two parameters were varied, namely, the reagent-to-pollutant ratio, i.e., weight of reagent/weight of WCB (g/g), and the ball-to-powder ratio, accounting for the different weights of balls used vs. weight of powder (powder refers to the sum of WCB and reagent). The substances selected as reagents were calcined CaO, dried marble sludge, and calcined marble sludge.

For the process being carried out, we can define two important parameters. The first one is the milling intensity (*I*), which is the rate of energy transferred to milled powder:

(12) $$I=\frac{1}{2}m_{b}v_{i}^{2}NF$$

The milling intensity is the kinetic energy (1/2*m_b_v_i_*^2^) delivered to the powder through the N balls (*m_b_* is the mass of each ball) by *F* hits per time unit. The units in SI are Watts. In the present work, all runs have been performed using the same rotational speed and number of balls in such a way that the values of *v_i_* and *F* should be constant among runs. Considering the calculations given by Concas et al. [11], the average velocity of the balls can be considered close to $v_{i}$ = 4.169 m/s, with the frequency of the impacts being *F* = 142.04 Hz.

The second important parameter is the energy dose, defined as the total amount of mechanical energy transferred to powder. Usually, this energy is defined as a specific quantity of energy per unit of powder mass, namely the specific dose (*D*), as follows:

(13) $$D=\frac{I\cdot t}{m_{p}}$$

i.e., *D* is the intensity transferred for a milling time ‘*t*’ to an *m_p_* amount of powder. Their units in SI are Joules/kilogram of powder (J/kg).

Considering the work by Delogu et al. [27] and Cagnetta et al. [28], two kinds of trends of the conversion expressed as a function of the specific energy dose can be found. On the one hand, conversion during the milling process (*X*) can be assimilated into an exponential model:

(14) $$X=1-\exp\left(-K_{E}D\right)$$

where *K_E_* is the rate constant of the exponential model (kg J^−1^ or s^2^·m^−2^).

On the other hand, a deeper study of the process suggests a sigmoidal model, expressed by the equation:

(15) $$X=1-\left(1+K_{S}\right)\exp\left(-K_{S}D\right)$$

where *K_S_* is the rate constant of the sigmoidal model (kg J^−1^ or s^2^·m^−2^).

The value of the term $\left(K_{S}\cdot D\right)$ was modified by Cagnetta [28], introducing the reagent ratio ‘*R*’, in order to account for the mass of powder consisting of reagent, obtaining a final equation such as:

(16) $$X=1-\left(1+RK_{S}D\right)\exp\left(-RK_{S}D\right)$$

where *R* = m_reagent_/m_WCB_. In the same way, the exponential model can be modified, introducing the reagent ratio in Equation (14):

(17) $$X=1-\exp\left(-RK_{E}D\right)$$

The calculation procedure consists of the optimization of the value of the constants (*K_E_* or *K_S_*, with different values for each of the three solids studied) for a particular set of runs, considering the following objective function, similar to Equation (11):

(18) $$OF=\sum_{all\;runs}^{}{\left(X_{experimental}-X_{calculated}\right)}^{2}$$

where *X_experimental_* is the experimental value of the conversion (i.e., debromination efficiency) and *X_calculated_* is the conversion value calculated by one of the previous models. The experimental debromination efficiency was determined through the analysis of the bromine content in the solid sample by ion chromatography and considering a minimum portion extracted for the analysis. In this case, the solid sample was solved in water by ultrasonic treatment, filtered, and then analyzed. The majority of the bromine content is transferred to the aqueous phase as bromide ions.

In the equipment used in this work, values of *v_i_* (m/s) and *F* (Hz) are taken as the ones proposed by Concas et al. [11]. In all the runs performed, the same rotational speed and mass of balls (7 balls of 13.9 g each) were used, and the intensity of the impacts is calculated by:

(19) $$I=\frac{1}{2}m_{b}v_{i}^{2}NF=\frac{1}{2}13.9\cdot 10^{-3}\;\mathrm{kg}\cdot {\left(4.169\right)}^{2}{\left(\frac{m}{s}\right)}^{2}\cdot 7\cdot 142.04\;\mathrm{Hz}=117.6\;W$$

This value equals 1234 W/g of balls. For the calculation of the specific dose, the value of intensity is multiplied by time (s) and divided by the total amount of powder. In this way, the specific dose of the different runs performed is calculated and shown in Table 5, together with other details of the performed runs. During the mechano-chemical treatment, as the reaction progresses, the reactants reduce in size because of the mechanical stresses to which they are subjected and are activated in turn (usually at the contact surface) to be able to react. Thus, thanks to the breakages and erosion provided (energy), the reactants undergo structural and chemical changes (formation of free radicals), which allow them to react with each other.

**Figure 5:**
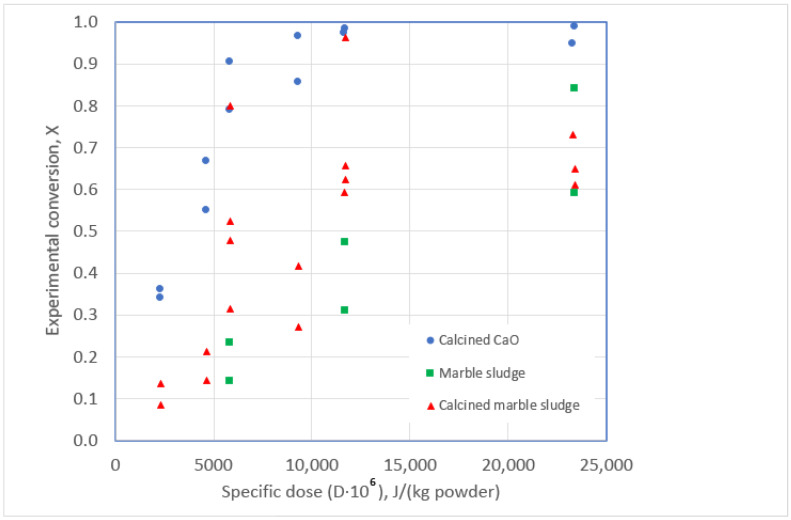
Debromination conversion vs. specific energy dose for the debromination of WCBs using different solid reagents.

In the runs performed, three different reagents were used, namely calcined CaO, dried marble sludge, and calcined marble sludge, as indicated in Table 5. Figure 5 shows the behavior of the process when studying the conversion obtained for the different specific doses with the three reagents studied. As expected, an increase in the specific dose results in an increase in conversion. The runs performed with CaO seem to be more effective for the debromination since, for a given value of D, the conversion values are higher with this reagent.

**Figure 6:**
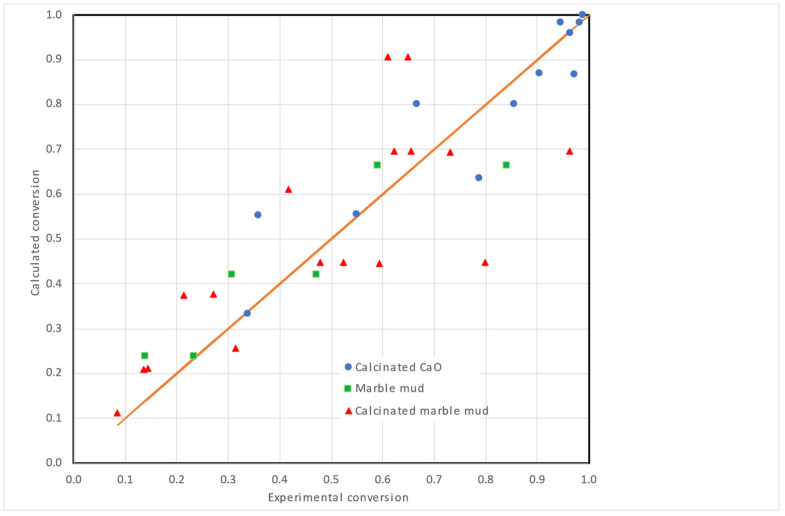
Calculated vs. experimental debromination conversion for different reagents and conditions.

Both exponential and sigmoidal models were used to correlate the experimental results, obtaining a slightly better fit using the exponential model for the debromination using all three reagents. Values of the coefficient of determination (R^2^) were 0.8779 for the exponential model and 0.8560 for the sigmoidal model. In Figure 6, the calculated vs. experimental values of the conversion are shown using the optimized values of the constants presented in Table 5.

Bearing in mind that all runs have been performed using the same rotational speed and number of balls, the values of *v_i_* and *F* should be constant among runs, so the relative values of $K_{E}^{\;}$ represent the efficiency of the reagent. As we can see, the most effective is CaO with a *K_E_* = 4.30 · 10^−8^ kg/J. Bearing in mind the other values of *K_E_*, the activity of the marble sludge is approximately 13% of that presented by CaO, but the marble sludge is quite activated by slight calcination, reaching *K_E_* the value of 1.6 × 10^−8^ kg/J, which is a 29% that of CaO. More temperature and time should be needed in order to decompose the CaCO_3_ present in the form of calcite in the marble sludge [29].

The values of the kinetic constants *K_S_* calculated by Cagnetta et al. [28] are approximately 100 times higher than the values of *K_E_* calculated in the present work. These can be considered as expected values because similar conversion values are obtained in the present work and that of Cagnetta and coworkers, but the milling times are much higher in our runs. It is also true that Cagnetta studied the debromination of a brominated reagent (hexachlorobenzene, HCB) directly added to the reagent used (calcined CaO), with which the access of CaO to the brominated molecules is much faster and more effective. Those authors also used much higher reagent ratios. Furthermore, the use of waste, such as marble sludge, is an environmentally friendly practice that should be valued, as the reuse of waste is part of the so-called circular economy.

## 4. Conclusions

The kinetics of the debromination of waste circuit boards (WCBs) was studied using two different pieces of equipment with different reaction mechanisms.

On the one hand, runs performed in water with K_2_CO_3_ at high temperature and pressure were explained by a combined model considering both mass transfer and chemical reaction. The model was able to fit runs performed for the debromination in pressurized reactors (reaction in liquid phase) at 200–225 °C. Comparing the rates of mass transfer and chemical reaction, we concluded that the mass transfer is much fater, both for both small and larger particles. In this sense, the process is governed by the superficial chemical reaction of removing bromine from the polymeric matrix.

In the planetary ball mill (reaction in solid phase), WCB is reacted with basic materials such as CaO and marble sludge (both natural and calcined). In these runs, the specific energy dose to which the material is subjected (J/kg) had a noticeable influence on the conversion obtained in the debromination reaction. For the kinetic study of the process, the modeling considered a single solid-reaction stage, showing a good fit of the experimental results to an exponential model. The model can predict conversion in different experimental situations and shows that CaO is much more active than marble sludge, but the activity of this material can be increased by the calcination of the reagent.

## Figures and Tables

**Table 1:** Runs performed in the HTC reactor for 2 h at the indicated temperature; experimental conditions and debromination efficiency (DE) [9]. Small particles of approx. 0.84 mm × 0.84 mm. The volume of liquid was, in all cases, 0.025 L.

Run No.	WCB Mass (g)	K_2_CO_3_ Mass (g)	Anionic CO_3_^2−/^Br^−^ Ratio	Solid/Liquid Ratio	T (°C)	% DE Achieved
1	4.9994	0.0145	1:25	1:5	200	3.26
2	4.9988	0.0073	1:50	1:5	200	2.65
3	5.0002	0.0363	1:10	1:5	200	6.25
4	2.5000	0.0073	1:25	1:10	200	3.00
5	2.4992	0.0036	1:50	1:10	200	2.68
6	5.0001	0.0354	1:10	1:5	200	5.36
7	4.9992	0.0721	1:5	1:5	200	4.67
8	4.9996	0.1457	1:2.5	1:5	200	12.79
9	9.9992	0.0291	1:25	1:2.5	200	3.68
10	10.0000	0.0159	1:50	1:2.5	200	3.04
11	4.9992	0.3631	1:1	1:5	200	42.33
12	4.9996	0.7253	2:1	1:5	200	51.04
13	4.9992	1.4499	4:1	1:5	200	53.09
14	9.9995	0.0298	1:25	1:1	200	4.96
15	9.9991	0.0153	1:50	1:1	200	5.12
16	12.4994	0.0358	1:25	1:2	200	3.55
17	12.5005	0.3635	1:2.5	1:2	200	30.72
18	12.5005	0.9061	1:1	1:2	200	49.99
19	12.4996	1.8122	2:1	1:2	200	65.75
20	12.4994	3.6246	4:1	1:2	200	74.23
21	4.9994	0.0146	1:25	1:5	225	12.37
22	5.0000	0.0075	1:50	1:5	225	9.82
23	4.9995	0.0364	1:10	1:5	225	13.59
24	2.5005	0.0072	1:25	1:10	225	9.35
25	2.5007	0.0038	1:50	1:10	225	8.47
26	12.4995	0.0359	1:25	1:2	225	10.51
27	12.4997	0.3618	1:2.5	1:2	225	58.14
28	12.5009	0.9061	1:1	1:2	225	92.29
29	12.4997	1.8132	2:1	1:2	225	99.38
30	12.4996	3.6246	4:1	1:2	225	99.58

**Table 2:** Runs performed in the HTC reactor at 225 °C; experimental conditions and debromination efficiency (DE) [30]. Pieces of approx. 8 × 3.3 cm.

Run No.	WCB Mass (g)	K_2_CO_3_ Mass (g)	Volume (L)	Anionic CO_3_^2−/^Br^−^ Ratio	Solid/Liquid Ratio	Time (h)	% DE Achieved
31	9.9092	0.7100	0.02	1:1	1:2	2	17.50
32	9.8161	0.7100	0.02	1:1	1:2	3	23.30
33	9.9979	0.7100	0.02	1:1	1:2	4	30.00
34	9.9678	0.7100	0.02	1:1	1:2	5	34.10
35	9.8895	0.7100	0.02	1:1	1:2	2	15.00
36	9.7287	0.7100	0.02	1:1	1:2	3	21.60
37	10.0260	0.7100	0.02	1:1	1:2	4	23.30
38	10.0233	0.7100	0.02	1:1	1:2	5	31.60
39	10.1384	1.4200	0.02	2:1	1:2	0.5	2.20
40	10.0113	2.8401	0.02	4:1	1:2	0.5	2.30
41	10.1082	1.4200	0.02	2:1	1:2	1	6.70
42	10.2577	2.8401	0.02	4:1	1:2	1	8.10
43	9.6430	1.4200	0.02	2:1	1:2	1.5	15.60
44	10.2186	2.8401	0.02	4:1	1:2	1.5	17.40
45	9.9253	1.4200	0.02	2:1	1:2	2	19.10
46	9.8963	2.8401	0.02	4:1	1:2	2	24.30
47	10.1759	1.4200	0.02	2:1	1:2	3	21.60
48	10.1940	2.8404	0.02	4:1	1:2	3	26.20
49	10.0416	0.7100	0.01	2:1	1:1	3	14.20
50	10.0756	1.4202	0.01	4:1	1:1	3	18.40
51	10.0442	1.4200	0.02	2:1	1:2	4	27.20
52	9.8975	2.8404	0.02	4:1	1:2	4	33.80
53	9.9930	0.7100	0.01	2:1	1:1	4	19.80
54	10.3819	2.8408	0.02	4:1	1:2	3	26.70
55	9.8675	4.2612	0.02	6:1	1:2	3	29.80
56	10.3407	2.8408	0.02	4:1	1:2	4	33.20
57	10.1579	4.2612	0.02	6:1	1:2	4	33.00
58	9.6174	2.8408	0.02	4:1	1:2	5	44.60
59	10.0881	4.2612	0.02	6:1	1:2	5	43.60
60	9.8860	2.8408	0.02	4:1	1:2	6	48.20

**Table 3:** Optimized values of the kinetic constants for liquid–solid debromination.

Constant	Runs	Optimized Value
*k_C_*_0_ (s^−1^ K^−1^)	1–30	1.49 × 10^5^
*k*_0_ (L (s g)^−1^)	(small particles)	8.2263
*k_C_*_0_ (s^−1^ K^−1^)	31–60	1.35 × 10^5^
*k*_0_ (L (s g)^−1^)	(larger pieces)	1.039
E/R (K^−1^)	1–60	3455

**Table 4:** Values of the kinetic constants for liquid-solid debromination at 225 °C.

Constant	Runs	Optimized Value
*k_C_* (s^−1^)	1–30	7.43 × 10^7^
*k*·C_Kmean_ (s ^−1^)	(small particles)	0.2792
*k_C_* (s^−1^)	31–60	6.73 × 10^7^
*k*·C_Kmean_ (s^−1^)	(larger pieces)	0.035

**Table 5:** Details on the runs performed in the planetary ball mill [29], kinetic constants, and calculated conversion.

Run No.	WCB Particle Diameter (mm)	Reagent Used	Ball to Powder Ratio (g/g)	Time (h)	Initial WCB (g)	Reagent Weight (g)	Experimental Conversion	D·10^−6^ Specific Dose (J/kg Reagent)	R (Reagent to Pollutant Ratio) (g/g)	*K_E_* 10^8^ (kg/J)	Calculated Conversion
2	Fine powder	Calcined CaO	20	2.5	0.95	3.81	0.340	2334	4	4.30	0.332
4	Fine powder		20	5	0.95	3.81	0.550	4668	4		0.554
6	Fine powder		20	10	0.95	3.81	0.857	9336	4		0.801
8	Fine powder		50	2.5	0.38	1.53	0.789	5816	4		0.635
10	Fine powder		50	5	0.38	1.53	0.974	11,633	4		0.867
12	Fine powder		50	10	0.38	1.53	0.946	23,266	4		0.982
14	Fine powder		20	2.5	0.53	4.24	0.359	2329	8		0.552
16	Fine powder		20	5	0.53	4.24	0.667	4658	8		0.799
18	Fine powder		20	10	0.53	4.24	0.966	9316	8		0.960
20	Fine powder		50	2.5	0.21	1.69	0.904	5847	8		0.868
22	Fine powder		50	5	0.21	1.69	0.984	11,694	8		0.983
24	Fine powder		50	10	0.21	1.69	0.989	23,388	8		1.000
26	Fine powder	Marble sludge	50	2.5	0.21	1.69	0.140	5847	8	0.577	0.238
28	Fine powder		50	5	0.21	1.69	0.309	11,694	8		0.419
30	Fine powder		50	10	0.21	1.69	0.591	23,388	8		0.663
32	0.84 × 0.84		50	2.5	0.21	1.69	0.234	5847	8		0.238
34	0.84 × 0.84		50	5	0.21	1.69	0.472	11,694	8		0.419
36	0.84 × 0.84		50	10	0.21	1.69	0.842	23,388	8		0.663
1	Fine powder	Calcined marble sludge	20	2.5	0.95	3.81	0.085	2334	4	1.26	0.112
3	Fine powder		20	5	0.95	3.81	0.143	4668	4		0.211
5	Fine powder		20	10	0.95	3.81	0.271	9336	4		0.377
7	Fine powder		50	2.5	0.38	1.53	0.315	5816	4		0.256
9	Fine powder		50	5	0.38	1.53	0.594	11,633	4		0.447
11	Fine powder		50	10	0.38	1.53	0.730	23,266	4		0.694
13	Fine powder		20	2.5	0.53	4.24	0.135	2329	8		0.210
15	Fine powder		20	5	0.53	4.24	0.213	4658	8		0.376
17	Fine powder		20	10	0.53	4.24	0.418	9316	8		0.610
19	Fine powder		50	2.5	0.21	1.69	0.478	5847	8		0.448
21	Fine powder		50	5	0.21	1.69	0.656	11,694	8		0.696
23	Fine powder		50	10	0.21	1.69	0.649	23,388	8		0.907
25	Fine powder		50	2.5	0.21	1.69	0.523	5847	8		0.448
27	Fine powder		50	5	0.21	1.69	0.623	11,694	8		0.696
29	Fine powder		50	10	0.21	1.69	0.611	23,388	8		0.907
31	0.84 × 0.84		50	2.5	0.21	1.69	0.799	5847	8		0.448
33	0.84 × 0.84		50	5	0.21	1.69	0.962	11,694	8		0.696
35	0.84 × 0.84		50	10	0.21	1.69	1.008	23,388	8		0.907
